# The Role of Extracellular Matrix and Hydrogels in Mesenchymal Stem Cell Chondrogenesis and Cartilage Regeneration

**DOI:** 10.3390/life12122066

**Published:** 2022-12-09

**Authors:** Magdalena Strecanska, Lubos Danisovic, Stanislav Ziaran, Michaela Cehakova

**Affiliations:** 1 National Institute of Rheumatic Diseases, Nabrezie I. Krasku 4, 921 12 Piestany, Slovakia; 2 Institute of Medical Biology, Genetics, and Clinical Genetics, Faculty of Medicine, Comenius University, Sasinkova 4, 811 08 Bratislava, Slovakia; 3 Department of Urology, Faculty of Medicine, Comenius University, Limbova 5, 833 05 Bratislava, Slovakia

**Keywords:** mesenchymal stem cells (MSCs), chondrogenesis, articular cartilage, extracellular matrix (ECM), hydrogels, regenerative medicine

## Abstract

Diseases associated with articular cartilage disintegration or loss are still therapeutically challenging. The traditional treatment approaches only alleviate the symptoms while potentially causing serious side effects. The limited self-renewal potential of articular cartilage provides opportunities for advanced therapies involving mesenchymal stem cells (MSCs) that are characterized by a remarkable regenerative capacity. The chondrogenic potential of MSCs is known to be regulated by the local environment, including soluble factors and the less discussed extracellular matrix (ECM) components. This review summarizes the process of chondrogenesis, and also the biological properties of the ECM mediated by mechanotransduction as well as canonical and non-canonical signaling. Our focus is also on the influence of the ECM’s physical parameters, molecular composition, and chondrogenic factor affinity on the adhesion, survival, and chondrogenic differentiation of MSCs. These basic biological insights are crucial for a more precise fabrication of ECM-mimicking hydrogels to improve cartilage tissue reconstruction. Lastly, we provide an overview of hydrogel classification and characterization. We also include the results from preclinical models combining MSCs with hydrogels for the treatment of cartilage defects, to support clinical application of this construct. Overall, it is believed that the proper combination of MSCs, hydrogels, and chondrogenic factors can lead to complex cartilage regeneration.

## 1. Introduction

**Figure 1:**
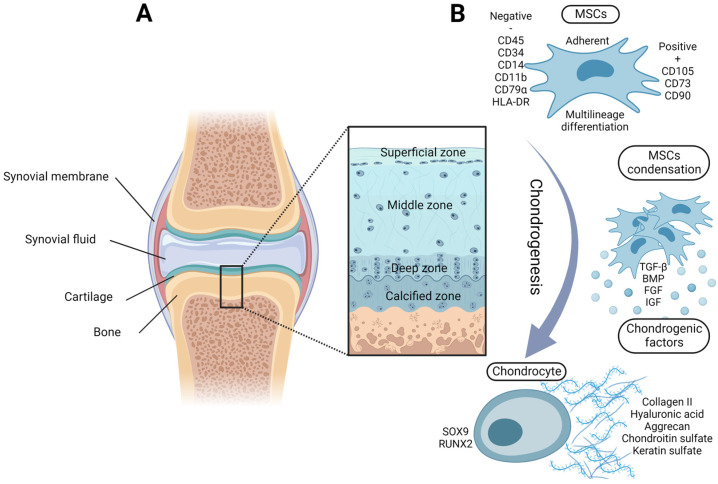
(**A**) A schematic illustration of the anatomical structure of articular cartilage with 4 histological zones. Joint tissue and articular cartilage are composed of chondrocytes and the extracellular matrix (ECM). (**B**) Chondrocytes of articular cartilage differentiate from mesenchymal stem cells (MSCs) that are characterized by high surface adhesion, a specific immunophenotype, and multilineage differentiation capacity. The process of chondrogenesis involves MSC condensation and environmental chondrogenic factors that cause the activation of transcription factors SOX9 and RUNX2. Differentiated chondrocytes then produce the cartilage-specific ECM. BMP: bone morphogenic protein; CD: cluster of differentiation; FGF: fibroblast growth factor; HLA-DR: human leucocyte antigen DR; IGF: insulin growth factor; RUNX2: runt-related transcription factor 2; SOX9: transcription factor SRY-box 9; TGF-β: transforming growth factor-beta2.

Articular cartilage, with a total thickness of 2 to 4 mm, provides a smooth and lubricated surface that prevents bone friction of diarthrodial joints and protects the subchondral bone. It consists of four histological zones (superficial, middle, deep, and calcified) (Figure 1A) created by chondrocytes and the extracellular matrix (ECM). Chondrocytes, the only cell type found in healthy cartilage, show high metabolic activity and produce cartilage-specific ECM molecules, such as collagen II type (COL2), hyaluronic acid (HA), aggrecan (ACAN), and chondroitin sulfate (CS). The cross-linking of these proteins and glycosaminoglycans (GAGs), by either physical or chemical bonds, results in the formation of a 3D network specific for the connective tissue of articular cartilage. Overall, chondrocytes represent only 2% of the cartilage structure. The remaining volume is filled by the ECM that retains high amounts of water. In addition, articular cartilage belongs to unique tissues with no blood, as well as no lymphatic or neural system, which markedly limits its regenerative potential. Nevertheless, chondrocytes can survive under harsh hypoxic conditions, but they completely depend on the passive diffusion of nutrients from the synovial fluid [1].

Cartilage remodeling refers to a common physiological phenomenon associated with the degradation of the old ECM and the synthesis of a new one. Maintaining the balance between these two processes is crucial for cartilage homeostasis [1]. Pro-inflammatory cytokines, such as interleukins (IL) IL-1β, IL-6, IL-8, and tumor necrosis factor-alpha (TNF-α), which are frequently enriched in the inflammatory environment, may disrupt this homeostasis. Due to the action of pro-inflammatory mediators, the expression of ECM-degrading enzymes (MMP-1, MMP-3, MMP-9, and MMP-13) is upregulated, leading to cartilage injury. This pathological situation is typical for inflammatory joint diseases, including osteoarthritis (OA) and rheumatoid arthritis (RA) [2]. The irreversible process of joint tissue degradation can cause subchondral bone damage, loss of joint function, and permanent disability [3,4]. Moreover, the full-thickness cartilage defects that reach the subchondral bone often result in the formation of mechanically insufficient COL1-abundant fibrocartilage [5,6]. The socio-economic burden of arthritic diseases, linked to costly treatment and disability of patients, is enormous [7]. The latest estimates suggest an increase in prevalence over time that will automatically lead to higher demand for medical interventions [8,9]. The traditional anti-rheumatic drugs, such as corticosteroids, nonsteroidal anti-inflammatory drugs (NSAIDs), and disease-modifying anti-rheumatic drugs (DMARDs, e.g., methotrexate), offer only pain relief and inflammation suppression [10]. On the other hand, the renewal of cartilage tissue using approaches of regenerative medicine can improve the symptoms and thus improve the patient’s quality of life. Currently, mesenchymal stem cells (MSCs) are becoming an attractive tool for cartilage tissue engineering considering their paracrine, reparative, immunomodulatory, and anti-inflammatory properties [11]. In this review, we provide insight into the role of the ECM and ECM-mimicking hydrogels in MSC chondrogenesis and cartilage regeneration offering evidence also from preclinical studies.

## 2. Chondrogenic Potential of Adult MSCs

Since the discovery of MSCs within the bone marrow in 1966 [12], these fibroblast-like cells have been also identified in other adult tissues and fluids, such as adipose tissue [13], synovial fluid [14], synovial membrane [15], and easily accessible urine [16]. The unlimited proliferation and multilineage differentiation capacity are typical features of MSCs. The International Society for Cell Therapy defined these minimal criteria for multipotent MSCs: (I) substrate/plastic adherence; (II) specific phenotype—the expression of CD105, CD73, and CD90, and at the same time lack of expression of CD45, CD34, CD14, CD11b, CD79a, and human leucocyte antigen DR (HLA-DR); (III) and the ability to differentiate into adipocytes, chondrocytes, and osteocytes [17]. The multilineage differentiation potential of MSCs, their presence in multiple adult tissues, their easy isolation and expansion potential in vitro, and their low immunogenicity make them an ideal candidate for clinical application in the field of regenerative medicine [18].

Physiologically, chondrocytes of articular cartilage differentiate from MSCs localized in the perichondrium, a thin fibrous coverage of cartilage [19,20]. The process of chondrogenesis includes MSC condensation, proliferation, and subsequent differentiation [21]. Therefore, the high-density 2D culture or micro-mass 3D culture are commonly used for the in vitro induction of MSC chondrogenesis together with chondrogenic differentiation factors such as insulin–transferrin–selenium, dexamethasone, L-proline, ascorbic acid phosphate, and transforming growth factor-beta (TGF-β) [22,23,24]. In addition to TGF-β, bone morphogenic proteins (BMPs), fibroblast growth factors (FGFs), and insulin growth factors represent other well-known signaling molecules involved in chondrogenesis [25]. Chondrogenic signaling leads to the activation of the transcription factors SRY-box 9 (SOX9) and runt-related transcription factor 2 (RUNX2) [26]. The expressions of SOX9 and RUNX2, along with COL2 and ACAN, are typical chondrogenic markers (Figure 1B). On the other hand, chondrocytes differentiated in vitro from MSCs can undergo hypertrophic differentiation. This process is associated with increased cell volume, matrix mineralization, COL10 expression, and bone formation impairing articular cartilage homeostasis [27,28].

MSCs from various sources differ in availability and chondrogenic potential. In stem cell research, bone-marrow-isolated MSCs (BMSCs) are still considered the gold standard and can be isolated from arthritis patients during joint surgery. Surprisingly, no difference in chondrogenic potentials was observed between BMSCs from healthy individuals and patients with OA/RA [29]. Neither the patient’s age nor the OA etiology has an impact on BMSC yield, proliferation, and chondrogenic differentiation capacity [30]. Similarly, adipose tissue is another important source of MSCs (ATSCs). ATSC isolation, however, requires liposuction and the cells possess lower chondrogenic potential compared to BMSCs [31]. The chondrogenic induction of both BMSCs and ATSCs leads to ECM formation containing GAGs but also the hypertrophic COL10 [32,33]. Hence, the identification of hypertrophy factors is important in MSC-mediated cartilage regeneration. For instance, the administration of the Rac-1 inhibitor was shown to prevent the in vitro and in vivo hypertrophic differentiation of ATSC-derived chondrocytes [34]. When it comes to MSCs derived from synovial fluid (SF-MSCs) and synovial membrane, these exhibit higher chondrogenic potential compared to BMSCs [35,36]. Synovial MSCs can be harvested during routine arthrocentesis in patients with arthritis [37]. Of note, OA synovial fluid contains a higher proportion of MSCs compared to healthy synovial fluid. Nonetheless, the presence of chronic inflammation impairs the therapeutic potential of SF-MSCs derived from long-standing RA patients [38]. Several subpopulations of synovial MSCs positive for CD73, CD39, and CD105 were identified to have superior chondrogenic potential even if isolated from patients with OA. Additionally, CD105^+^ synovial MSCs exclusively form the hyaline-type cartilage without the presence of COL1 [39,40]. Finally, another population of stem cells with great chondrogenic potential, tested both in vitro and in vivo, is represented by urine-derived stem cells (UdSCs). The non-invasive isolation of UdSCs most frequently requires up to 200 mL of freshly voided urine. Due to their easy and low-cost method of isolation, UdSCs attract much attention as a potential cell source for regenerative medicine [41,42]. Kidneys are the most likely source of UdSCs. This hypothesis is supported by the presence of the Y-chromosome in UdSCs isolated from female patients, who underwent kidney transplantation from male donors. Comparing the proliferation capacity, UdSCs from healthy donors are superior to BMSCs, but dispose with the lower chondrogenic potential. However, the UdSC-laden decellularized articular cartilage ECM efficiently stimulated the healing process of knee cartilage in rabbits and no significant difference in cartilage regeneration was detected between UdSCs and BMSCs [43]. Additionally, the UdSC-derived ECM supports the chondrogenic differentiation of BMSCs in late passages and increases the capacity to improve osteochondral defects in vivo in synovial MSCs [44,45].

## 3. Involvement of ECM Physical Properties and Signaling in MSC Chondrogenesis

**Figure 2:**
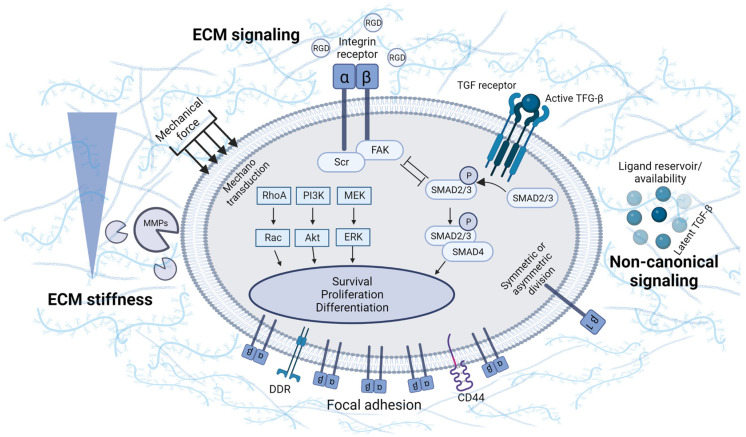
Cellular processes can be modulated by the extracellular matrix (ECM). ECM stiffness and integrin signaling, which is triggered upon integrin interaction with the ECM, are transmitted through integrin/FAK signaling and mechanosensitive pathways. In addition, individual ECM components affect the availability and activity of signaling molecules (e.g., TGF-β), which induces non-canonical signaling. Overall, the ECM influences cellular survival, proliferation, and differentiation. CD: cluster of differentiation; DDR: discoidin domain receptor; FAK: focal adhesion kinase; MMPs: matrix metalloproteinases; RGD: arginine–glycine–aspartic acid sequence; TGF: transforming growth factor.

Initially, the ECM was considered to be more of a scaffold, which provided an area for cells to adhere and concentrate. Hynes´s statement: "ECM not just pretty fibrils" captures the current knowledge about the ECM. Nowadays, it is well known that the ECM considerably affects cell behavior and promotes cell adhesion, migration, proliferation, and differentiation. Biological processes can be modulated by the ECM, either directly through physical properties and molecular composition, or indirectly via the activation of cellular signaling [46] (Figure 2). MSCs themselves form a specific ECM, thus creating a unique supportive environment. As an example, the co-culture of BMSCs with a BMSC/ATSC-derived ECM enhances proliferation and prevents spontaneous differentiation. For that reason, it is suitable for the long-term in vitro expansion of MSCs [47]. Furthermore, the stimulatory effect of the ECM on MSC chondrogenic differentiation has been described using the ECM from decellularized cartilage [48,49].

The extracellular matrix is known to have a porous structure and tissue-specific stiffness. The pore size regulates the cellular shape, differentiation, and secretion. Matrices with pore sizes ranging from 94 to 300 µm were created using a freeze-drying technique. BMSCs cultured in the 300 µm matrix showed improved proliferation, chondrogenic differentiation, and ECM production [50]. Additionally, the matrix with a pore size smaller than 125 µm promotes chondrogenesis while maintaining the differentiated chondrocyte phenotype of articular chondrocytes [51]. The stiffness of the ECM can be also transmitted to cells as a mechanical signal that is sensed by several mechanosensitive pathways such as Ras/MAPK, PI3K/Akt, Rho/ROCK, and Wnt/β-catenin [52,53]. The conversion of a mechanical signal to a biochemical one is executed by YAP/TAZ transcription factors. These are known to mediate MSC differentiation in response to ECM stiffness [54]. Importantly, cartilage destruction, associated with ECM degradation and reduced stiffness, can thus cause an insufficient activation of these mechanosensitive pathways. The stiffness of healthy articular cartilage, defined by the modulus of elasticity, corresponds to 0.2–1.1 MPa. It has been reported that substrates with stiffness in this range induce the chondrogenic differentiation of chondroprogenitor ATDC5 cells. Furthermore, the 0.5 MPa substrate triggers the expression of SOX-9, COL2, and ACAN, therein resembling the effects of TGF-β, and concurrently upregulates the mRNA level of TGF-β. In 0.5 MPa gels, the autocrine TFG-β1 production was shown to be mediated by the ROCK pathway [55]. Thus, the substrate stiffness controls the differentiation fate of TGF-β-stimulated MSCs. The cultivation of TGF-β-treated MSCs in a soft substrate leads to the induction of chondrogenic markers while preventing the assembly of α-actin stress fibers typical for myogenesis [56]. Low-stiffness substrates induce the expression of the SOX-9 early marker. On the other hand, stiffer substrates induce RUNX-2 expression, which is involved in terminal differentiation. As the MSC chondrogenesis progresses with time, the substrate is required to become stiffer [57]. However, a higher expression of the COL10 in MSCs seeded on stiffer substrates has been identified as an unwanted effect [58]. In fact, not only substrate stiffness, but also ECM components, such as HA, regulate the chondrogenic process in MSCs. The physical and biochemical properties of the ECM are difficult to separate as the stiffness of the ECM increases with the increasing concentration of the ECM. Gels with constant stiffness can be prepared by the special modification of the ECM. The cultivation of ATSCs in a constant low-stiffness gel resulted in a higher expression of ACAN and COL2 after the addition of HA molecules. Conversely, the addition of HA to stiffer gels reversed the chondrogenic induction of ATSCs, indicating a nonlinear relationship between ECM composition and stiffness on chondrogenesis [59].

MSCs form physical connections with the ECM, referred to as focal adhesions. The cell–ECM interaction is primarily mediated through integrin receptors, but also molecules other than integrin, such as the discoidin domain receptor (DDR) and the CD44 receptor, are able to bind the ECM [60,61]. Integrin signaling is triggered by the activation of heterodimeric transmembrane receptors composed of alpha and beta subunits. Integrin receptor activation leads to conformational changes, which, in turn, cause focal adhesion kinase (FAK) and SH2 kinase recruitment and activation. The main downstream signaling pathways activated by integrin signaling are MAPK/ERK, PI3K/Akt, and small GTPases of the Rho family that regulate cell survival, proliferation, migration, and differentiation [62]. A micro-mass culture of chicken MSCs, used for the MSCs tests of chondrogenic differentiation, resulted in FAK activation [63]. Likewise, MSCs seeded on a fibroblast-derived matrix displayed higher FAK expression [64]. It has also been evidenced that the association of FAK with β1 integrin is downregulated by MMP-2. Thus, MMP-2 acts as a negative regulator of chondrogenic condensation. It is noteworthy that MMP-2 is present at high concentrations in patients with OA, which can, at least partially, explain the mechanism behind impaired chondrogenesis [65,66]. The homeostasis of the symmetric and asymmetric division of MSCs is affected by the β1 integrin subunit as well [67]. Moreover, the weak interaction of fibronectin with α5 integrin is linked to the absence of chondrogenic differentiation in MSCs, despite being cultured in chondrogenic media [68]. Zhang et al. describe an interesting negative reciprocal interaction of TGF-β and integrin/FAK signaling in the regulation of MSC chondrogenesis and hypertrophy. The initial chondrogenic induction of MSCs in free-swelling conditions enhances the expression of β1 integrin and activation of FAK/ERK signaling. The subsequent exposure to dynamic compression suppresses integrin/FAK/ERK signaling and hypertrophy while maintaining the activity of TGF-β signaling [69].

As mentioned above, the ECM can also modulate cellular signaling indirectly by so-called non-canonical signaling. Several signal proteins can directly bind to the ECM, which serves as a reservoir for growth factors and influences their availability and activity [46]. For example, ECM proteins regulate the activity of the chondrogenic factor TGF-β. This growth factor is secreted by several cell types and immediately forms a latent complex with latent TGF-β binding protein (LTBP). The LTBP couples TGF-β to fibrillin and fibronectin molecules within the ECM. TGF-β activation is subsequently accompanied by the degradation of ECM-binding proteins or conformational alterations in the latent complex [70]. The interaction between the VWC domain of the COL2 protein (isoform A) and TGF-β1 or BMP-2 was also described [71]. The alternative splicing of the COL2 gene in mature cartilage leads to the loss of the VWC domain and may thus weaken the binding ability of chondrogenic transcription factors [72]. In addition, heparin and heparan sulfate in the structure of ECM proteoglycans have a high affinity for FGF and serve as co-activators of FGF receptors [73].

## 4. Hydrogels

Nowadays, it is common practice to synthesize artificial scaffolds which mimic the biological, chemical, and physical properties of the articular cartilage ECM. Hydrogels represent a 3D network of hydrophilic polymers with a porous structure that swells in an aqueous environment [74]. Hydrogels must possess biological attributes that support the transport of nutrition, cell adhesion, viability, proliferation, migration, and differentiation. From a chemical point of view, hydrogel requirements need to meet the concept of non-toxicity, biocompatibility, and biodegradability. These materials must also fulfill the load parameters of the original tissue, especially in the case of weight-bearing joints. The complex regenerative strategy of joint tissue very often relies on three variables: hydrogel, chondrogenic growth factors, and MSCs. Despite promising results of various tested combinations, the induction of the regenerative process of articular cartilage is still a subject of ongoing research. Since 1990, a wide spectrum of hydrogel-forming polymers, both natural and synthetic, has been described [75]. The following text will further discuss individual types of natural, synthetic, injectable, and functionalized hydrogels. We will also provide evidence about MSC-laden hydrogels with a focus on articular cartilage regeneration.

### 4.1. Natural Polymers for Hydrogels

Many natural polymers, including collagen/gelatin, HA, and chondroitin sulfate, can also be found in native articular cartilage and cleaved by naturally occurring enzymes, ensuring high biocompatibility and biodegradability. Moreover, the presence of RGD sequence (e.g., tripeptide: Arg-Gly-Asp) in the structure of several natural polymers supports integrin-mediated cellular attachment to the hydrogel [76]. Chitosan and alginate are non-cartilaginous polymers that typically form hydrogels with weaker mechanical properties and are frequently used as a component of the polymer blend [74].

HA is a linear polysaccharide composed of dihydrate units of glucuronic acid and N-acetylglucosamine. It is also the most abundant GAG of articular cartilage involved in joint lubrication. Chondrocytes can directly interact with HA through surface receptors CD44 and CD168. High levels of CD44 were also identified in ATSCs. Moreover, in ATSCs cultured on an HA-coated plate, the chondrogenic differentiation was enhanced [77]. Inversely, blocking the HA receptors results in a lower expression of COL2 and ACAN in MSCs seeded on HA hydrogels [78]. A major disadvantage of HA-based hydrogels is their enzymatic in vivo degradation by the action of hyaluronidases. Although, a lower expression of hyaluronidases 2 and 3 was observed in MSCs encapsulated in HA hydrogels cultured in a chondrogenic medium, compared to a growth medium [79]. In different animal models of osteochondral defects, the MSC-laden HA hydrogel is the most frequently preclinically tested type of polymer [80]. Lee et al. confirmed that the intra-articular injection of BMSCs together with HA supported the healing of cartilage in minipigs with osteochondral damage [81]. Similarly, the administration of bone marrow aspirate in combination with HA led to complete coverage of full-thickness cartilage defects in goats [82]. Chiang et al. evaluated the effect of the BMSCs/HA construct in a rabbit model of OA. A better histological cartilage score and less cartilage loss were reported in the BMSC/HA-treated group compared to the HA and control groups [83]. The administration of BMSCs/HA also resulted in superior clinical and radiological outcomes in donkeys with different degrees of OA [84]. Another scientific team compared the therapeutic effects of BMSC and HA alone or in combination. Only the BMSC/HA combination increased COL2 production and promoted a successful histological repair of damaged cartilage in pigs with OA [85]. The safety and short-term efficacy of intra-articular administrated BMCSs and HA were also clinically validated in patients suffering from knee osteochondral defects [86]. Moreover, the combination of the HA hydrogel with ATSCs slowed OA progression in sheep. Additionally, an 18-week retention of ATSCs was reported after their intra-articular administration [87]. Besides the slower progression of OA, lower levels of inflammatory factors in synovial fluid of the ATSCs/HA-treated group in comparison to the HA group were measured by the same research team [88]. In addition, the injection of UdSCs from healthy volunteers combined with HA mediated more efficiently in vivo neocartilage formation compared to other groups (UdSCs w/o HA, pure HA, and saline). This regenerative effect was confirmed by an improved histological score as well [41].

Collagens are triple-helical proteins highly abundant in the skin, bones, cartilage, and tendons. Despite the natural presence of COL2 in native cartilage, its administration may have an atherogenic effect. Hence, it is commonly used for the experimental modeling of arthritis in mice [89]. That is why COL1 is favored for hydrogel preparation in cartilage regeneration. UdSCs cultured on COL1-coated plates show greater proliferative capacity and an ability to preserve stem cell characteristics [90]. COL1 was also proven to support chondrogenic induction and ACAN expression in BMSCs [91]. However, when comparing the impact on chondrogenic potential, COL2 is superior to COL1 [92]. Administrating collagen-based hydrogel laden with ATSCs improved cartilage regeneration in a rabbit model of OA [93]. Moreover, COL2-enriched hydrogels induce the condensation of MSCs via the α10 integrin. In comparison, MSCs cultured in denatured COL2 hydrogels have reduced condensation and GAG production [94].

Gelatin is a single-strand derivate created by the denaturation of native collagen. When cultured in gelatin and under chondrogenesis-stimulating conditions, ATSCs form numerous aggregates of spheroid morphology. These structures were found to be positive for GAGs, contrasting ATSCs cultured on plastic only [95]. In addition, gelatin elevates the level of GAGs in ATSCs during the initial days of culture compared to synthetic polymers [96]. Currently, one preclinical study confirmed the therapeutic effectiveness of gelatin-based hydrogels laden with resveratrol-treated BMSCs in a rabbit osteochondral model. This combination led to the formation of a hyaline cartilage with a high expression of chondrogenic markers COL2 and GAGs [97].

Chondroitin sulfate (CS) is a natural polysaccharide present in the ECM structure of cartilage. The injectable CS/polyethylene glycol (PEG) hydrogel promotes chondrogenic differentiation and, at the same time, reduces the production of pro-inflammatory cytokines IL-6 and IL-1β in encapsulated ATSCs. This CS/PEG hydrogel also mimics the cartilage structure, promotes the survival of ATSCs, and the production of GAGs [98]. Moreover, enhanced aggregation and chondrogenic differentiation of BMSCs, as evidenced by COL2, ACAN, and SOX9 expression, were reported in cells seeded on CS/PEG hydrogel. Conversely, lower levels of COL1 and COLX were observed in BMSCs cultured on PEG/CS hydrogels compared to PEG hydrogel only, suggesting the role of CS in blocking chondrocyte hypertrophy [99].

Chitosan is a well-known chitin derivative that is naturally distributed within the exoskeleton of many invertebrates. It is composed of randomly arranged glucamine and N-acetylglucosamine residues. Structurally, this linear polysaccharide resembles the GAGs of articular cartilage. The in vivo application of chitosan material is suitable for the regeneration of osteochondral defects and leads to the formation of hyaline-type cartilage [100]. Chitosan-based hydrogels have been confirmed to support the survival, proliferation, and chondrogenic differentiation of encapsulated MSCs [101]. COL2-enriched chitosan hydrogels with RGD sequence increase the attachment of MSCs and the expression of COL2 and ACAN [102]. Moreover, a histological improvement in the osteochondral defect in vivo was observed using a combination of injectable chitosan-based hydrogel and SF-MSCs [103].

### 4.2. Synthetic Polymers for Hydrogels

ECM-mimicking synthetic polymers usually show better mechanical properties, while lacking biocompatibility and biodegradability. The best-known polymers include PEG, poly(vinyl alcohol) (PVA), poly(lactic acid) (PLA), poly(glycolic acid) (PLG), and their co-polymer poly(lactic-co-glycolic acid) (PLGA). The degradation of synthetic polymers is mediated by the hydrolysis of covalent bonds [104]. Despite its synthetic nature, a blend containing PLA promotes chondrocyte adhesion, survival, and proliferation [105]. PLA/PVA gels also support higher chondrogenesis compared to polymer-free conditions [106]. Concurrently, PEG gel supports the aggregate formation and COL2/ACAN expression of encapsulated BMSCs [107]. To support cellular attachment, bio-inert synthetic polymers require functional modification with RGD motifs or blend formation. To illustrate, compared to unmodified PEG hydrogels, PEG hydrogel enriched with an adhesive RGD sequence supports the viability and chondrogenic differentiation of encapsulated MSCs [108]. Additionally, the combination of PEG gel with COL1 and COL2 results in higher GAG production in encapsulated MSCs [109]. Synthetic PEG diacrylate/HA hydrogel containing encapsulated MSCs derived from arthroscopic synovial fluid promotes the healing of damaged cartilage in rats [110]. The administration of PLGA-PEG-PLGA hydrogel loaded with BMSCs also alleviated joint inflammation and improved cartilage reconstruction in mice with RA [111].

### 4.3. Injectable and Functionalized Hydrogels

**Figure 3:**
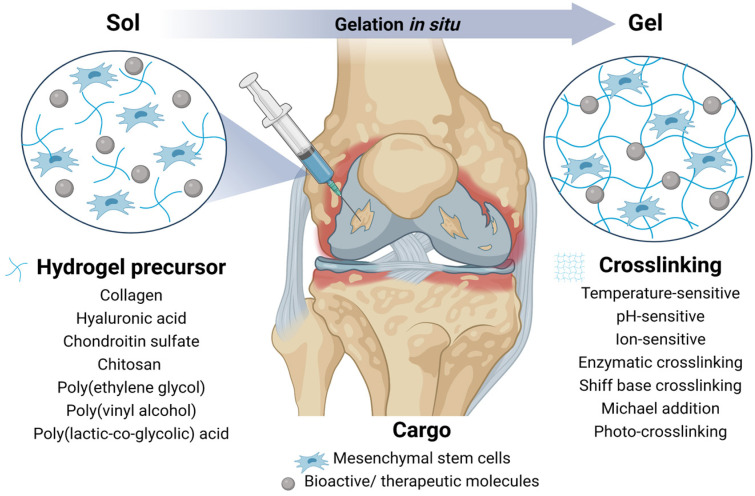
Injectable hydrogel for articular cartilage regeneration. A liquid mixture of hydrogel precursor, mesenchymal stem cells, and bioactive molecules can be easily injected into the damaged site of cartilage. The administration is followed by polymer crosslinking and in situ gelation.

Injectable hydrogels are capable of sol–gel transition and in situ gelation. Easy injection delivery to the site of damaged cartilage makes them minimally invasive and prevents risks associated with surgical procedures. In fact, injectable hydrogels also allow for irregular cartilage erosion sites to be filled better and for bioactive molecules to be added [112] (Figure 3). In situ gel formation utilizes the hydrogel’s sensitivity to temperature, pH, and ion concentration changes. The spontaneous crosslinking of sensitive hydrogels occurs after their delivery to a site with a physiological temperature (37 °C) [113], pH (7.4) [114], or in the presence of Ca^2+^ [115]. Another group of injectable polymers depends on chemical crosslinking mediated by enzymes, Schiff base, Michael addition, and photo-crosslinking. This topic is summarized in a review by Liu et al. Chemical methods usually require polymer modification and polymerization initiators [116].

For instance, injectable methacrylate–gelatin (crosslinked by visible light) promotes a long-term culture of BMSCs, induces chondrogenesis, and GAG production more efficiently compared to agarose-encapsulated BMSCs [117]. Injectable hydrogels can be easily functionalized with bioactive substances that directly support the chondrogenesis of MSCs. The addition of TGF-β1 (5 ng/scaffold) to injectable heparin/HA hydrogel and its uniform release seem to be crucial for the induction of chondrogenesis of encapsulated chondroprogenitor cells [118]. Alternatively, platelet lysate can serve as a more accessible but less specific source of growth factors. Its chondroinductive potential was confirmed in BMSCs cultured in the platelet lysate-supplemented HA hydrogel [119]. Kartogenin is another currently tested small bioactive molecule that mediates RUNX1 activation and COL2 and ACAN expression, therein triggering MSC chondrogenesis [120]. The hostile RA joint microenvironment, with the over-production of pro-inflammatory cytokines and reactive oxygen species, steered the fabrication of a drug-loaded hydrogel. The local application of these “self-healing” hydrogels can reduce serious adverse effects and enhance the low efficacy of common anti-rheumatic drugs. Recently, Zhao et al. tested the therapeutic potential of infliximab HA hydrogel (integrated into a printed porous scaffold) implanted to a rabbit model of RA. The combination of a porous scaffold, infliximab (1 mg/mL), and 10^5^ of ATSCs decreased the level of pro-inflammatory cytokines and mediated in vivo cartilage and subchondral bone repair [121].

## 5. Conclusions

A better understanding of ECM-regulated pathways is essential to achieve a positive clinical outcome in patients with articular cartilage damage. It is even more important in the case of combined therapy using MSCs and ECM/hydrogels. Multiple pieces of evidence support the importance of ECM physical properties such as pore size and stiffness in the enhancement of MSCs chondrogenic potential and maintaining the differentiated chondrocyte phenotype. Moreover, the ECM has the capacity to mediate the mechanosensitive signaling and influence the availability and activity of chondrogenic factors. The proper knowledge of this complex ECM-mediated regulation helps to improve MSC chondrogenic maturation and functionality by complementing the process of chondrogenesis involving MSC condensation and chondrogenic factors. Insights into the biology of the ECM are also crucial for advancing the ECM-mimicking hydrogel fabrication to maximize the regenerative potential of MSCs and thus enhance the clinical benefits of MSC-loaded hydrogels in the treatment of articular cartilage defects. 

## Data Availability

Not applicable.
